# The role of kynurenine pathway and kynurenic aminotransferase alleles in postpartum depression following cesarean section in Chinese women

**DOI:** 10.1002/brb3.1566

**Published:** 2020-02-26

**Authors:** Chengxuan Quan, Saiying Wang, Kaiming Duan, Jiahui Ma, Heya Yu, Mi Yang, Na Hu, Ge Long, Guang Zeng, Zhendong Huang

**Affiliations:** ^1^ Department of Anesthesia The Third Xiangya Hospital of Central South University Changsha China; ^2^ Department of Anesthesia Changsha Taihe Hospital Changsha China; ^3^ Department of Anesthesiology Union Hospital Tongji Medical College Huazhong University of Science and Technology Wuhan China

**Keywords:** genetic variations, kynurenic aminotransferase, kynurenine pathway, postpartum depressive symptoms

## Abstract

**Objectives:**

A growing body of data indicates that the kynurenine pathway may play a role in the pathogenesis of postpartum depressive symptoms (PDS). Kynurenic aminotransferase (KAT) is an important kynurenine pathway enzyme, catalyzing kynurenine (KYN) into kynurenic acid (KYNA). This study investigated as to whether genetic variations in KAT are associated with PDS.

**Methods:**

A cohort of 360 Chinese women scheduled to undergo cesarean delivery was enrolled into this study. PDS was determined by an Edinburgh Postnatal Depression Scale (EPDS) score ≥ 13. A total of eight KAT single nucleotide polymorphisms (SNPs) were genotyped and their association with PDS investigated. Serum concentrations of KYN, KYNA, and quinolinic acid (QUIN) in women with or without PDS were also measured. This allowed the determination of the KYNA/KYN ratio, which is reflective of KAT activity.

**Results:**

Postpartum depressive symptoms incidence was 7.2%. Advanced maternal age, lower education, antenatal depression, and postpartum blues were risk factors for PDS (*p* < .05). Women with PDS, versus non‐PDS, had heightened KYN levels one day prior to surgery (ante‐d1) (*p* < .05), as well as having significantly lower KYNA and higher QUIN levels at postnatal day three (post‐d3) (*p* < .05). Women with, versus without, PDS also had a significantly higher QUIN/KYNA ratio at post‐d3 (*p* < .05). KAT activity was significantly lower in women with, versus without, PDS at ante‐d3 (*p* < .05). No significant association was evident between the KAT SNPs and PDS.

**Conclusion:**

Our data support a role for alterations in the kynurenine pathway in the pathogenesis of PDS, although no significant association was found for the eight tested KAT SNPs with PDS.

## Graphical abstract

Our data support a role for alterations in the kynurenine pathway in the pathogenesis of PDS, although no significant association was found for the eight tested KAT SNPs and PDS.

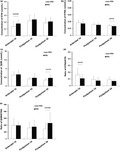

## 1 INTRODUCTION

Postpartum depressive symptomatology (PDS) is a depressive syndrome that affects 6.5%–19% of women following childbirth and is the most common mental health disorder following delivery (O'Hara & McCabe, 2013; Stewart & Vigod, 2016). PDS not only interfere in the parturient's everyday life, work, and marital/family relationships, but may also affect the infant's emotion and cognition function. PDS may also increase the risk of mental retardation and behavior changes in neonates as well as significantly heightening the offspring's risk of developing psychiatric disorders (Brummelte & Galea, 2016; Stein et al., 2014). Although progress has been made in identifying risk factors, pathogenesis, and treatment of PDS, its biological underpinnings of PDS still require clarification.

Kynurenine pathway was involved in many psychological diseases. Approximately 95% of tryptophan (TRP) is metabolized by the kynurenine pathway, with the remaining 5% of TRP metabolized by the serotonin/melatonin pathway (Maddison & Giorgini, 2015). In the kynurenine pathway, TRP is converted to kynurenine (KYN) by indoleamine 2,3‐dioxygenase (IDO) and/or tryptophan 2,3‐dioxygenase (TDO). KYN may then be converted to kynurenic acid (KYNA) by kynurenine aminotransferase (KAT) and to 3‐hydroxykynurenine (3‐HK) by kynurenine‐3‐monooxygenase (KMO). 3‐HK can be converted to further kynurenine pathway products, eventually leading to quinolinic acid (QUIN), which can be excitotoxic via the N‐methyl‐d‐aspartate (NMDA) receptor. The NMDA receptor is an important mediator of glutamatergic activity, including long‐term potentiation and long‐term depression, and therefore of processes important to learning and brain functioning. Heightened QUIN production and NMDA receptor overactivation may be involved in the pathogenesis of depression. In contrast, KYNA is an endogenous NMDA receptor antagonist, thereby acting to antagonize the excitotoxic effects of QUIN (Duan et al., 2018; Lau & Zukin, 2007; Schwarcz, Bruno, Muchowski, & Wu, 2012).

A growing body of data indicates that the kynurenine pathway plays an important role in PDS pathogenesis. Data indicate that an increased QUIN/KYNA ratio damages hippocampal neurons (Hanson, Owens, & Nemeroff, 2011; Zunszain et al., 2012), thereby correlating with depressive symptomatology. The recent meta‐analyses on KYN pathway in depression draw a conclusion that decreased levels of kynurenine are found in unipolar major depression versus healthy controls but studies are significantly heterogeneous in nature (Arnone et al., 2018). We have previously shown that the conversion of KYN to 3‐HK is increased in postpartum depressed mothers, suggesting that disturbance in the balance between KYNA and 3‐HK/QUIN may be important to the biological underpinnings of PDS (Wang et al., 2017). Kynurenine metabolites are likely to play a role in major depression, but an exact etiological role in mood disorder seems complex and requires further research.

Raised KYNA levels not only lower QUIN‐induced excitotoxicity but also QUIN production, with suppressed KYNA levels thereby elevating QUIN synthesis and associated excitotoxicity. Although other enzymes can modulate levels of KYNA synthesis, KAT activity is a major determinant of KYNA levels and thereby important to the etiology and course of a number of medical disorders, including depression (Han, Cai, Tagle, & Li, 2010). KAT is the main rate‐limiting enzyme in KYNA generation, with KAT blockers significantly determining cerebral KYNA levels in animal models (Nematollahi, Sun, Jayawickrama, Hanrahan, & Church, 2016). Unlike IDO and KMO, KAT is not induced by pro‐inflammatory factors, but can be regulated by pharmacological interventions and physical activity (Yu et al., 2004). Genetic factors can also influence the expression and activity of KAT, with the KAT gene C/T rs1480544 polymorphism decreasing immune function and influencing DNA repair (Boros, Bohár, & Vécsei, 2018). The KAT haplotype CGCGCT is more frequent in major depressive disorder, versus controls, especially for anxiety‐related symptomology (Claes et al., 2011). However, the relationship between KAT alleles as well as kynurenine pathway metabolism and the pathophysiology of PDS is unknown. On the basis of the association between the kynurenine pathway and PDS, and the role of KAT haplotypes in depression risk, we selected cesarean delivery mothers to explore the relationship between kynurenine pathway metabolites and KAT single nucleotide polymorphisms (SNP) distribution with PDS incidence.

## 2 MATERIALS AND METHODS

### 2.1 Participants and study design

After obtaining IRB approval (S155, Human Research Ethics Committee, the Third Xiangya Hospital of Central South University) and informed consent, 360 ASA I‐II, aged 18 years or older parturients, scheduled to undergo cesarean delivery from March 2015 to February 2017 were recruited to this study. Exclusion criteria were as follows: previous psychiatric history; important organ (including heart, brain, lung, liver, and kidney) dysfunction; psychoactive substance or alcohol abuse; previous antipsychotic treatment within a month prior to enrollment; uncooperative regarding the completion of questionnaires and related information (Pinsonneault et al., 2013).

### 2.2 Anesthesia protocol and management

Spinal anesthesia was used during the operation. Fifteen micrograms ropivacaine and 25 μg fentanyl diluted with 10% glucose 1 ml were injected into the subarachnoid space to reach an optimal level of anesthesia. Postoperative analgesia was provided as a standard practice in our department. Arterial blood pressure (BP) was controlled to within 25% of baseline (day before surgery) systolic value, while heart rate (HR) was controlled to 50 ~ 90 bpm, as appropriate. A blinded observer in the postanesthesia care unit (PACU) monitored the recovery indexes, such as agitation, anesthesia level, visual analogue scale (VAS) scores as well as postoperative nausea and vomiting (PONV) (Wille, 2004). After a one hour stay in the PACU, all patients were discharged to the ward, with any reported discomfort controlled.

### 2.3 Neuropsychology tests and general information collection

The Edinburgh Postpartum Depression Scale (EPDS), a self‐report, ten‐item questionnaire (including one on self‐harm), was administered to indicate neuropsychological testing at baseline (1 day before surgery) and at 3 days and 42 days after surgery. A total score of >14 was used to classify patients with antenatal depression (Gibson, McKenzie‐McHarg, Shakespeare, Price, & Gray, 2009). Scores greater than 9 and 12 were respectively used to classify postpartum blues (PB) or PDS (Gibson et al., 2009). All participants’ social demographic data were obtained prior to cesarean delivery. General information was also collected, including age, weight gain, term duration, primiparous or not, fetal gender, education level, family pregnancy planning, method of fertilization, occupation or not, family income, antenatal depressed, and postnatal blue.

### 2.4 Measurement the plasma concentrations of KYN, KYNA, and QUIN by HPLC

Blood samples were withdrawn in the morning in EDTA‐containing tubes at the following time points: before surgery, 1 day and 3 days after surgery. All parturients were normally required a fasting time of 8 hr prior to each blood draw. The samples were immediately transferred to the biomedical laboratory and centrifuged for 10 min at 3000‐g. Plasma was removed and stored at −80°C until analysis. Some whole blood samples collected before surgery were also stored at −80°C until analysis. Following sample quality control, data analysis was carried out on 24 participants with PDS and 48 participants without PDS. The plasma concentrations of KYN, KYNA, and QUIN in the two groups were measured by HPLC.

### 2.5 Selection of KAT SNP loci and genotyping

We chose the target SNP loci according to the 1,000 Genome Browser data and dbSNP database as well as previous research results that had clinical significance with KAT gene SNP loci. Every SNP locus had to satisfy a minor allele frequency (MAF) of greater than 5% in Southern Han Chinese. Hence, a total of eight KAT SNP loci were finally selected: KATI rs7046797; KATII rs17711677, rs2279267, rs4145964; KATIII rs12729558, rs11804245, rs14490, and rs3738055. Primer design was performed using Sequenom Assay Design 3.1 software based on SNP sites (Lee et al., 2014). Two microliter of each extension primer was drawn and mixed. Two microliter was drawn by pipette from the extension primer and mixed into 40 ul ddH_2_O for mass spectrometry.

### 2.6 Statistics

Statistical analysis was carried out with the SPSS 18.0 software package. All data were described as mean (standard deviation [*SD*]), percentage, and frequency. The variation in general sociodemographic and clinical data between PDS and non‐PDS parturients was analyzed using the chi‐square test. All successfully genotyped SNPs were tested for Hardy–Weinberg equilibrium prior to further chi‐square test to find the relationship between different genotypes and PDS (Shi & He, 2005). Student's *t* test was applied to detect the relationship of PDS incidence with serum KYNA, KYN, or QUIN concentration [KYNA], [KYN], or [QUIN], and its ratio, [KYNA]/[KYN] or [QUIN]/[KYNA], in PDS and non‐PDS parturients at three time points. A probability of *p* < .05 was used to determine significance.

## 3 RESULTS

### 3.1 General characteristics and PDS

The PDS incidence was 7.2% in the present sample. There were no significant differences in PDS, versus non‐PDS, patients regarding general characteristics such as weight gain, term duration, primiparity, fetal gender, family pregnancy planning, method of fertilization, occupation, and family income (*p* > .05) (Table 1). Advanced maternal age, lower education, antenatal depression, and PB were significant PDS risk factors (*p* < .05) (Tables 1 and 2).

**Table 1:** General characteristics of all participants

General characteristics		non‐PDS (frequency)	PDS (frequency)	*p*	OR (95%CI)
Age	≥35	43 (81.1%）	10 (18.9%)	.002	4.230 (1.803–9.921)
	<35	291 (94.8%)	16 (5.2%)		
Weight gain (kg)/ Mean (*SD*)		16.92 (5.200)	15.17 (4.619)	.097	
Full‐term pregnancy	Yes	308 (92.8%)	24 (7.2%)	.156	1.078 (1.046–1.111)
	No	26 (100%)	0 (0%)		
Primiparity	Yes	221 (93.6%)	15 (6.4%)	.381	1.434 (0.638–3.225)
	No	113 (91.1%)	11 (8.9%)		
Fetal gender	Female	157 (92.9%)	12 (7.1%)	1.000	
	Male	167 (92.8%)	13 (7.2%)		
	Both	10 (90.9%)	1 (9.1%)		
Educational level	Elementary education	93 (88.6%)	12 (11.4%)	.049	0.452 (0.202–1.014)
	Specialized education	240 (94.5%)	14 (5.5%)		
Planning of pregnancy	Yes	224 (92.9%)	17 (7.1%)	.976	0.987 (0.413–2.359)
	No	104 (92.9%)	8 (7.1%)		
Method of fertilization	Natural	305 (93.3%)	22 (6.7%)	.254	1.912 (0.162–1.563)
	Artificial	29 (87.9%)	4 (12.1%)		
Occupational	No	76 (91.6%)	7 (8.4%)	.627	0.800 (0.324–1.974)
	Yes	258 (93.1%)	19 (6.9%)		
Family income (yuan/month)	<5,000	88 (91.7%)	8 (8.3%)	.812	
	5,000–20,000	223 (92.9%)	17 (7.1%)		
	>20,000	21 (95.5%)	1 (4.5%)		
Antenatal depression	No	305 (96.2%)	12 (3.8%)	.000	12.270 (5.192–28.996)
	Yes	29 (67.4%)	14 (32.6%)		
Postnatal blues	No	293 (98.0%)	6 (2.0%)	.000	22.091 (8.348, 58.459)
	Yes	42 (68.9%)	19 (31.1%)		

**Table 2:** Multivariable analysis of general characteristics and the incidence of PDS

	*B*	*SE*	Wald	*df*	*p*	Exp(*B*)	95% CI of EXP(*B*)	
							Lower limit	Upper limit
Antenatal depression	0.914	0.441	4.305	1	.038	2.495	1.052	5.920
Postpartum blues	2.297	0.431	28.381	1	.000	9.945	4.271	23.153

### 3.2 Impact of AND and PB on PDS

**Figure 1:**
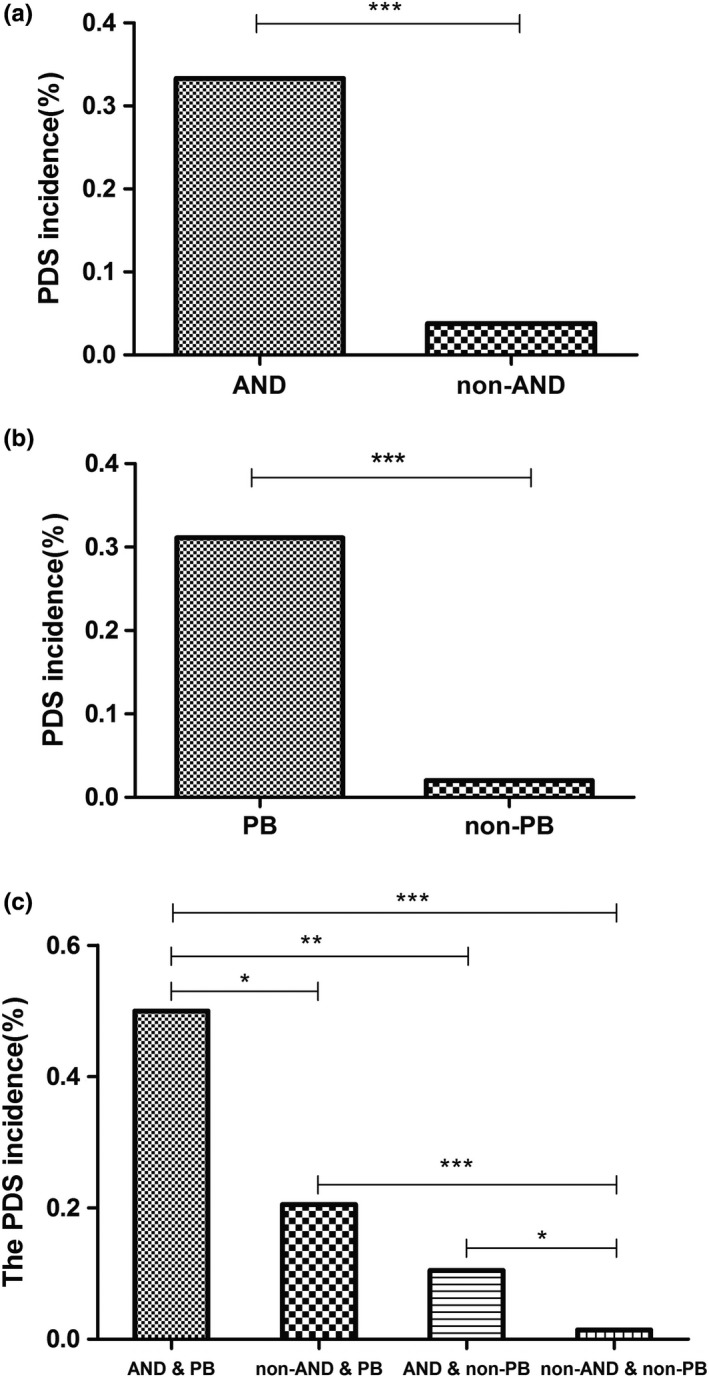
The impact of AND & PB on the PDS incidence. a and b show the PDS incidence difference between AND and non‐AND parturients and between PB and non‐PB parturients, respectively. c shows nearly half of AND & PB parturients suffers PDS. *p < .05, **p < .01, ***p < .001. AND, antenatal depression; PB, postpartum blues; PDS, postpartum depressive symptoms

The data indicate that women with antenatal depression (AND) and/or PB have a higher incidence of PDS. The incidence of PDS was as high as 33.3% in women with antenatal depression, versus 3.8% in women without antenatal depression (*p* < .001). Similarly, the PDS incidence was 31.1% in women with PB, versus 2.0% in women without PB (*p* < .001). When combining antenatal depression and PB, the incidence of PDS was 52.1%, compared with 1.4% in women with neither condition. The latter incidence was the lowest among the four subgroups (*p* < .05) (Figure 1).

### 3.3 Variation of plasma KYN, KYNA, and QUIN concentration

**Figure 2:**
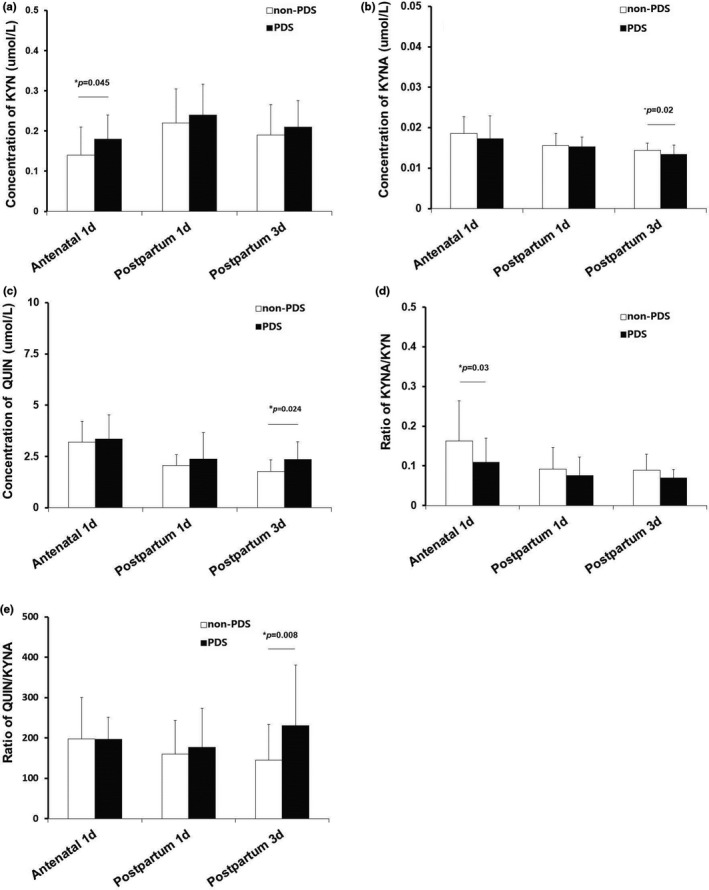
Peri‐partum plasma KYN, KYNA, and QUIN concentration and the variation of QUIN/KYNA and KYNA/KYN. (a) The serum KYN concentration of women with, versus without, PDS. (b) The serum KYNA concentration of women with, versus without. (c) The serum QUIN concentration of women with, versus without, PDS. (d) The ratio of KYNA/KYN of women with, versus without, PDS. (e) The ratio of QUIN/KYNA of women with, versus without, PDS. KYN, kynurenine; KYNA, kynurenic acid; PDS, postpartum depressive symptoms; QUIN, quinolinic acid

The serum KYN concentration of women with, versus without, PDS increased on the day before delivery (*p* < .05) (Figure 2a), which also increased on the 1st and 3rd day postpartum, although there was no statistical difference (*p* > .05) (Figure 2a). There were either no statistical differences between these two groups in serum KYNA concentration on antenatal day 1 and postpartum day 1 (*p* > .05) (Figure 2b). However, the serum KYNA concentration of women with, versus without, PDS on the 3rd day postpartum was significantly lower (*p* < .05) (Figure 2b). No statistical difference in serum QUIN concentration was found on the day before delivery and the 1st day postpartum between the two groups (*p* > .05) (Figure 2c). However, the serum QUIN concentration of women with, versus without, PDS on postpartum day 3 was significantly higher (*p* < .05) (Figure 2c).

### 3.4 Variation of QUIN/KYNA and KYNA/KYN

Women with, versus without, PDS showed a nonsignificant difference in the QUIN/KYNA ratio on antenatal day 1 and postpartum day 1 (*p* > .05) (Figure 2e), respectively. However, in women with, versus without, PDS the QUIN/KYNA ratio was significantly higher on postpartum day 3 (*p* < .05) (Figure 2e). Women with, versus without, PDS also showed a lower KYNA/KYN ratio on antenatal day 1 (*p* < .05) (Figure 2d). The KYNA/KYN ratio in women with PDS on the postpartum days 1 and 3 showed a nonsignificant difference (*p* > .05) (Figure 2d), respectively.

### 3.5 Variation of KAT gene and incidence of PDS

The KAT gene SNPs (rs17711677, rs2279267, rs4145964, rs12729558, rs11804245, rs14490, and rs3738055) were obtained by the gene chip method. The genotype distributions of these SNPs in PDS and non‐PDS group are in concordance with the Hardy–Weinberg equilibrium. The relationship between each SNP and the incidence of PDS is shown in Table 3. KAT SNP distributions, as well as the associations between KAT SNPs and PDS incidence, are shown in Table 3. None of the KAT alleles showed a significant association with PDS incidence (Table 3).

**Table 3:** Variation of KAT gene and incidence of PDS

Gene	SNP	Genotype	PDS (frequency)	Non‐PDS (frequency)	*p*
*KATⅠ*	rs7046797	CC	22 (7.2%)	283 (92.8%)	.961
		CT	3 (7%)	40 (93.0%)	
		TT	0 (0%)	1 (100%)	
*KATⅡ*	rs17711677	GG	18 (8.1%)	205 (91.9%)	.609
		GC	6 (5.2%)	109 (94.8%)	
		CC	1 (9.1%)	10 (90.9%)	
*KATⅡ*	rs2279267	AA	8 (5.7%)	133 (94.3%)	.521
		AG	15 (9.1%)	150 (90.9%)	
		GG	3 (7.0%)	40 (93.0%)	
*KATⅡ*	rs4145964	CC	9 (6.3%)	148 (93.7%)	.775
		CT	13 (8.3%)	144 (91.7%)	
		TT	3 (8.6%)	32 (91.4%)	
*KATⅢ*	rs11804245	TT	26 (7.6%)	316 (92.4%)	.39
		TG	0 (0%)	9 (100%)	
*KATⅢ*	rs12729558	CC	7 (6.6%)	99 (93.4%)	.502
		CG	11 (6.5%)	158 (93.5%)	
		GG	8 (10.5%)	68 (89.5%)	
*KATⅢ*	rs3738055	CC	7 (6.7%)	97 (93.3%)	.512
		CT	11 (6.5%)	158 (93.5%)	
		TT	8 (10.5%)	68 (89.5%)	
*KATⅢ*	rs14490	AA	19 (7.4%)	237 (92.6%)	.673
		AC	6 (6.8%)	82 (93.2%)	
		CC	1 (16.7%)	5 (83.3%)	

Note

There was no significant association between KAT gene variations and PDS.

### 3.6 Linkage disequilibrium and haplotype analyses

The linkage disequilibrium analysis of KAT gene SNPs rs17711677, rs2279267, and rs4145964 showed that *D*′ = 100, r^2^ = 86 in rs2297267 and rs4145964 (Table 3), indicating a strong linkage between the two loci. However, further haplotype analysis showed no relationship of PDS incidence with rs17711677 and rs2279267 (Table 4).

**Table 4:** Haplotype analysis between KATⅡ SNP loci

Loci	Case (frequency)	Control (frequency)	*p*	OR (95%CI)
CG	8 (16%)	117 (18.2%)	.663	0.841 (0.384–1.838)
GA	31 (62%)	404 (62.7%)	.797	0.925 (0.511–1.675)
GG	11 (22%)	112 (17.4%)	.447	1.311 (0.651–2.639)

The linkage disequilibrium analysis of KAT gene SNPs rs11804245, rs12729558, rs3738055, and rs14490 showed that *D*′ = 100, r^2^ = 100 in rs12729558 and rs3738055 (Table 3), indicating a strong linkage between the two loci. However, further haplotype analysis showed no relationship of PDS incidence with rs11804245 and rs3738055, rs14490 (Table 5).

**Table 5:** Haplotype analysis between KATIII SNP loci

Loci	Case (frequency)	Control (frequency)	*p*	OR (95%CI)
TCA	25 (48.1%)	345 (53.5%)	.380	0.777 (0.441–1.368)
TTA	19 (36.5%)	202 (31.3%)	.488	1.231 (0.684–2.218)
TTC	8 (15.4%)	87 (13.5%)	.743	1.140 (0.519–2.504)

## 4 DISCUSSION

The results of the current study showed: (a) Advanced maternal age, lower education, antenatal depression, PB, and inflammation were PDS risk factors; (b) antenatal depression and PB were the main PDS predictors; (c) the metabolites of kynurenine pathway changed over the perinatal period, and these changes were closely related to the emergence of PDS; (d) KAT activity in women with PDS was lower than that in women without PDS; (e) there was no significant relationship between KAT genetic variations and PDS.

A growing body of data supports a role for the kynurenine pathway in PDS (Oxenkrug, 2013). Clearly, the results indicate the relevance of changes in the kynurenine pathway in PDS pathophysiology, as indicated by comparisons in the levels and ratios of kynurenine pathway products in women with, versus without, PDS. This is supported by previous data (Veen et al., 2016; Wang et al., 2017).

Given that approximately 95% of TRP is utilized by the kynurenine pathway, it is clear that elevated levels of pro‐inflammatory cytokines and stress, by increasing IDO and TDO, can drive some of the remaining 5% of TRP down the kynurenine pathway and away from the serotonergic and melatonergic pathways (Anderson, 2018)^.^ The data in the present study show PDS to be associated with heightened kynurenine pathway activity in the postnatal period, as compared to antenatal day 1. It is also of note that women with PDS showed higher levels of kynurenine pathway activity at antenatal day 1, indicating heightened kynurenine pathway activity perinatally and possibly over the course of pregnancy. As stress and antenatal depression are risk factors for PDS, this could suggest that activated kynurenine pathway activity may contribute to poorer fetal and maternal outcomes in such pregnancies, which is supported by a wide array of preclinical studies (Hanson et al., 2011; Hanson et al., 2011; Nematollahi et al., 2016; Wang et al., 2017; Zunszain et al., 2012). This also links to our previous data showing heightened IDO levels in parturients with PDS (Duan et al., 2018)^.^

Although KYN may not be directly neuroactive, by acting as a precursor for the synthesis of KYNA and QUIN, it can have powerful and contrasting effects, on neuronal functioning, as indicated above (Hanson et al., 2011; Zunszain et al., 2012). The present results showed that levels of KYNA decreased in PDS, versus non‐PDS, parturients, with QUIN levels and the QUIN/KYNA ratio increasing in PDS. Previous studies indicate increased QUIN and decreased KYNA to be evident in depressed patients (Laugeray et al., 2010; Skalkidou, Hellgren, Comasco, Sylvén, & Poromaa, 2012). As KYNA can decrease QUIN synthesis and inhibit QUIN's excitotoxicity via the NMDA receptor (Goebel‐Goody et al., 2012), the present results suggest a role for heightened NMDA receptor activity in PDS, as has been proposed for depression more widely.

Kynurenic aminotransferase is the rate‐limiting enzyme in the catalysis of KYN to KYNA. KAT is therefore important to the regulation of KYNA levels, with both clinical and preclinical investigations using the KYNA/KYN ratio as a proxy of KAT activity (Han et al., 2010). The results of the current study indicate that maternal perinatal KAT activity is lower in PDS, versus non‐PDS, women. Some benefits of exercise may be mediated by increased KAT levels in muscles, leading to a heighted level of plasma KYNA and lower KYN levels (Claes et al., 2011). These authors suggest that the heightened conversion to KYNA, which does not cross the blood–brain barrier (BBB), may decrease the transfer of KYN, which does cross the BBB, and thereby lower the central conversion of KYN to 3‐HK and QUIN, and their associated neurotoxicity. As such, PDS pathoetiology may be partly mediated by changes in kynurenine pathway regulation systemically as well as centrally. This may be one way in which the weakening of perinatal KAT activity, systemically and/or centrally, may be involved in PDS pathophysiology. Clearly, this requires further investigation. As previous research indicates a role for genetic factors in PDS risk and KAT alleles in the modulation of general depression susceptibility, the present study also investigated the role of eight KAT alleles in PDS risk. However, no significant correlation of these eight KAT SNPs and PDS was evident. As the current, and previous, results indicate a role for the kynurenine pathway in PDS, it may require a larger sample size investigation to clarify the role, if any, of KAT alleles.

There is a growing appreciation that many of the effects of heightened kynurenine pathway activity are mediated by a decrease in the levels of serotonin and melatonin (Kim & Jeon, 2018; Li, Hu, Yang, Oxenkrug, & Yang, 2017). Decreased serotonin has long been associated with depression (Anderson, Jacob, Bellivier, & Geoffroy, 2016). However, there is a growing appreciation of the importance of serotonin as a precursor for N‐acetylserotonin and melatonin. Lower melatonin may be of particular importance pre‐ and perinatally, given its high production by the placenta and its role in optimizing mitochondria functioning, which has relevance to the pathophysiology of depression more widely. It may also be of note that higher levels of kynurenine may activate the aryl hydrocarbon receptor, which is an important modulator of immune system‐patterned activity, especially as the aryl hydrocarbon receptor has been implicated in depression and may interact with melatonin and different kynurenine pathway products on the mitochondria outer membrane (Anderson & Rodriguez, 2015). The role of such interactions in the linking and differentiation of antenatal and postnatal depression as well as PB will be important to determine in future studies.

The research also finds several PDS risk factors. Advanced maternal age is a risk factor for PDS since older women may bring more obstetrical complications during delivery. Educational level may influence parturient's personalized psychological conditions which further exerts impacts on PDS. Data in the present study clearly show that antenatal depression and PB are important PDS risk factors, which is similar with previous researches that approximately half of women with antenatal depression and PB eventually develop PDS (Grigoriadis et al., 2019). More risk factors may be found in further researches.

The shortcomings of this study are its short‐term, cross‐sectional design. Future studies will benefit from a longitudinal design, including the assessment of psychological status during pregnancy and the postpartum period as well as from the determination of metabolites of the kynurenine pathway and their interaction with other factors linked to depression. A larger sample size may be required to determine the role of KAT alleles in the pathophysiology of PDS. It is also of note that cesarean section may be associated with an increase in the levels of pro‐inflammatory cytokines that switch off maternal pineal gland melatonin synthesis, leading to a circadian dysregulation, which is an area of much interest in the pathophysiology of mood disorders more widely. As such, the investigation of differences in women receiving a cesarean section, versus those with a vaginal birth, will be important to determine.

In summary, this study investigated PDS occurrence in parturients that underwent cesarean section and the association of the kynurenine pathway and KAT alleles with PDS. Our findings indicate that PDS is associated with decreased KAT activity and an increased QUIN/KYNA ratio, although no significant relationship was found between KAT alleles and PDS. The present results also indicate antenatal depression and PB to be powerful risk factors for PDS.

## CONFLICT OF INTEREST

The authors declare no conflicts of interest.

## Data Availability

Data available on request from the authors (Quan et al., 2020).
